# Effect of gold nanoparticles shape and dose on immunological, hematological, inflammatory, and antioxidants parameters in male rabbit

**DOI:** 10.14202/vetworld.2022.65-75

**Published:** 2022-01-19

**Authors:** Eman T. Mehanna, Basma S. A. Kamel, Dina M. Abo-Elmatty, Sameh M. Elnabtity, Manal B. Mahmoud, Mostafa M. Abdelhafeez, Ahmed Sabry S. Abdoon

**Affiliations:** 1 Department of Biochemistry, Faculty of Pharmacy, Suez Canal University, Ismailia, Egypt; 2 Pharmacist at Ministry of Health, Zagazig, Al-Sharqia, Egypt; 3 Department of Pharmacology, Faculty of Veterinary Medicine, Zagazig University, Zagazig, Egypt; 4 Department of Immunology, Animal Reproduction Research Institute, ARC, Haram, Giza, Egypt; 5 Department of Food Science and Technology, Faculty of Agriculture, Misurata University, Libya; 6 Department of Animal Reproduction and Artificial Insemination, Veterinary Research Division, National Research Center, Dokki, Cairo, Egypt.

**Keywords:** antioxidant’s activity, dose, gold nanorods, gold nanospheres, hematological, immunological parameters, rabbit

## Abstract

**Background and Aim:**

Gold nanorods (AuNRs) have gained much attention recent years due to their promising optical and chemical properties and are hence used in applied research and industrial nanotechnology. This study was designed to investigate the effect of gold nanoparticle shape (Gold nanorods vs. gold nanosphere) on immune response in rabbit.

**Materials and Methods:**

Thirty New Zealand white rabbits were divided into six groups (n=5 rabbits). The first group is the control negative received an intravenous (IV) injection of normal saline 0.9%; the second group (vaccinated) is the control positive, and the other four groups were vaccinated and received a single-dose or repeated five consecutive IV doses of 300 mg/kg body weight 50 nm AuNRs or 50 nm gold nanosphere (50 nm AuNSs) dissolved in ultrapure water. Blood and serum were collected for the hematological and biochemical analysis.

**Results:**

White blood cells (WBCs) count, lymphocytes, monocytes, eosinophils, and basophils showed significantly (p<0.05) higher values with the repeated-dose AuNRs. g-globulin levels showed a significant difference after 15 days in the single-dose AuNSs. Single-dose AuNSs significantly (p<0.05) increased the immunoglobulin G (IgG) and significantly (p<0.05) decreased the tumor necrosis factor-alpha. In addition, it elicited a significant (p<0.05) decrease in the malondialdehyde levels and a significant (p<0.05) increase of the superoxide dismutase, glutathione peroxidase, and catalase levels. Moreover, evoked red blood cells count, mean corpuscular volume, and mean corpuscular hemoglobin were significantly (p<0.05) lower than the control group. The platelet count, lysozymes, and nitric oxide were significantly (p<0.05) higher in repeated-dose AuNRs.

**Conclusion:**

The effect of AuNPs is shape and dose-dependent. The repeated 5 days IV 50 nm AuNRs doses over 15 days showed a significant antioxidant effect, with no considerable toxicity or vascular reactions.

## Introduction

Nanotechnology is a growing field of study focused on the development of nanomaterials less than 100 nm, and various sizes, shapes, chemical compositions, controlled dispersity, and their potential use for human benefits [1]. Moreover, nanoparticles contain molecular fluorophores, which make them ideal probes for biodiagnostic applications [2].

Of the many forms of nanoparticles, gold nanoparticles (AuNPs) are the most clinically useful ones [3]. AuNPs have significant exploitations in the biomedical field due to (1) their comparative chemical stability, making them less hazardous, (2) simple and straightforward synthesis and fabrication process, and (3) genuine biocompatibility and non-interference with other labeled biomaterials (e.g., antibody and other biomarkers) [4].

The shape of metal nanoparticles plays an important role in modulating their optoelectronic and catalytic properties [5]. Recent studies indicated that AuNPs of 25–50 nm in diameter are the optimal size for cellular uptake and efficiently stimulate membrane wrapping and receptor-ligand interaction to drive the NPs into the cell [6]. AuNPs also exhibit different shapes such as spherical, sub-octahedral, octahedral, decahedral, icosahedral multiple twined, multiple twined, irregular shape, tetrahedral, nano triangles, nanoprisms, hexagonal platelets, and nanorods [7]. Gold nanorods (AuNRs) have gained much attention in the recent few years due to their specific optical and chemical property and hence they were used in biological applications [8]. AuNRs have various applications in *in vivo* imaging due to the plasmon resonance absorption and scattering of light in the near-infrared region [9].

Moreover, AuNPs have been used in biomedical sciences, including imaging, genomics, biosensors, immunoassays, clinical chemistry, detection and control of microorganisms, rheumatoid arthritis [10], cancer cell phototherapy [11], targeted delivery of genes/drugs, or other substances [12]. Noteworthy is that AuNPs are being increasingly administered to animals and humans parentally. However, their impact on human and environmental health remains unclear.

Despite the *in vitro* studies, there are few toxicologic reports to characterize the toxicity of AuNPs using animal models [13]. The *in vivo* toxicity includes many parameters such as dose, route of exposure, metabolism, excretion, and immune response. The toxicological profiles of nanomaterials might also be determined by nanomaterial chemical composition, size, shape, aggregation, and surface coating [13].

Therefore, this study aims to compare the effects of gold nanosphere (AuNSs) or AuNRs on the hematological and immunological parameters and antioxidants activity in rabbits. The effect of AuNP shape on the immune response has been previously explored. Therefore, the present study examined for the 1^st^ time the effect of different shapes and the effect of single or repeated injection of AuNPs on blood picture, immune-modulatory markers, and the oxidative stress parameters in the rabbit.

## Materials and Methods

### Ethical approval

The study was conducted in accordance with the requirement of the Institutional Animal Care Committee and was reviewed and approved by the Ethical Committee, Faculty of Pharmacy, Suez-Canal University, Egypt (201804BHDA1).

### Study period and location

This study was conducted from September 2018 to September 2020 at the Experimental Animal House, Faculty of Veterinary Medicine, Zagazig University.

### Synthesis and characterization of 50 nm AuNRs

AuNRs solution was prepared using the seed growth approach according to the method adopted by Murphy *et al*. [14]. The absorption spectra of AuNRs solutions were determined using a V-630 ultraviolet (UV)–visible spectrophotometer (Jasco, Japan). A strong absorption band with a maximum at ~808 nm resulting from the electronic oscillation of the electrons of the nanorod along its long axis and a weak band at ~530 nm polarized along the short axis resulting from nanorod electrons oscillations along the short axis of the nanorod. Transmission electron microscopic (TEM) images were obtained using JEOL JEM 2010 TEM operated at 200 kV accelerating voltage.

### Synthesis and characterization of 50 nm AuNSs

Fifty nanometers AuNSs were prepared using the citrate reduction method according to the method adopted by Frens [15]. For the preparation of 50 nm AuNPs, in a clean conical flask, add 100 mL aqueous solution of 0.25 mM HAuCl_4_ and heat to boiling with stirring. After that 3.5 mL of 1%, aqueous solution (wt/v) of sodium citrate was added. Heating was maintained until a deep ruby red color was developed, indicating the formation of AuNPs. The boiling and stirring were extended for 30 min and then cooled at 25ºC. Transmission electron microscopy and UV–visible spectrometry measurements were used to characterize the prepared AuNSs.

### Experimental design

Thirty New Zealand white rabbits of 2-3 months old and weighing about 1.8-2 kg were used. Rabbits were divided into six groups (n=5 rabbits). The first group (G1) serves as a negative control (non-treated and non-vaccinated) and received intravenous (IV) injection of normal saline 0.9% and the second group (G2) serves as control positive and was vaccinated with polyvalent rabbit Pasteurella vaccine. The other four groups (G3=Single-dose AuNSs, G4=Single-dose AuNRs, G5=5 days repeated-dose AuNSs, and G6=5 days repeated-dose AuNRs) were vaccinated and received an IV single-dose or repeated five consecutive doses 1 mL of 300 mg/kg body weight (BWT) 50 nm AuNRs or 50 nm AuNSs dissolved in ultrapure water over 5 days [13].

On days 1, 8, and 15 post-treatment, blood samples from each group were collected on an ethylenediaminetetraacetic acid for hematological study. Plan blood samples were also collected for serum separation. Serum samples were used for biochemical parameters including antioxidant enzymes (superoxide dismutase [SOD], catalase [CAT], and glutathione peroxidase [GPx]) and lipid peroxidation marker (malondialdehyde [MDA]), as well as the immunological parameters. Regarding the nitric oxide and lysozyme, the serum was separated from blood samples on days 1, 2, and 3 post-treatment.

### Immunological and inflammatory parameter analysis

The complete blood count with white blood cells (WBCs) differentials was done using Sysmex® autoanalyzer (Chennai, Tamil Nadu, India). The lysozyme in the serum at the first few days (1^st^, 2^nd^, and 3^rd^ days) post-treatment was measured according to the method described by Schultz [16].

Regarding the lymphocyte transformation test on the 8^th^ day post-treatment, the separation of lymphocytes was performed according to the method of Boyum [17]; then, total lymphocyte count was performed according to Hudson and Hay [18], after that, the preparation of mitogens was according to Rai-El-Balahaa *et al*. [19]; finally, the Modified Masson’s Trichrome staining was conducted according to the method of Denise *et al*. [20]. The IgG in the serum on the 15^th^ day post-treatment was measured according to the method described by Erhard *et al*. [21].

Qualitative fractionation of serum proteins to determine serum gamma-globulins was carried out using polyacrylamide gel columns according to the technique described by Daves [22].

The tumor necrosis factor-alpha (TNF-α) in the serum on the 15^th^ day post-treatment was measured according to the method described by Olaniyan *et al*. [23]. The comet assay was carried out according to the technique described by Singh *et al*. [24].

### Biochemical analysis

For biochemical analyses, kits were purchased from Biodiagnostic Company, Egypt. Oxidative stress parameters**:** Nitric oxide level was measured during the first 3 days after treatment (day 1-3 post-treatment) according to the method described by Ramadan and Attia [25]. MDA concentration was determined according to the method previously described [26]. CAT activity was determined following the method previously described [27]. GPx catalyzes the following reaction with glutathione reductase (EGPx-100, Pascual *et al*. [28]). SOD test was done according to Nishikimi *et al*. [29].

### Statistical analysis

Data were analyzed using the Statistical Package for the Social Sciences (IBM Corp., NY, USA). Results of the biochemical and immunological estimations were presented as mean±standard deviation of mean. The total variation was analyzed by performing a one-way analysis of variance. p≤0.05 was considered statistically significant based on Tukey’s honestly significant difference.

## Results

### Synthesis and characterization AuNRs and AuNSs

**Figure-1:**
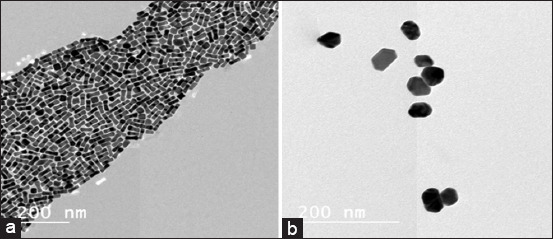
Photomicrograph using transmission electron microscopic showing 50 nm gold nanorods (a) and 50 nm gold nanosphere, (b) (bar=200 nm).

The morphology and size of synthesized AuNRs and AuNSs were determined using TEM. The images clearly showed that the average size of AuNRs was found to be about 50.1±8.2 nm and they were relatively homogeneous in diameter and rod in shape (Figure-1a). TEM images of 50 nm AuNSs illustrate a uniform sphere shape of AuNSs with regular distribution; the diameter of AuNPs was 50 nm (Figure-1b).

### Effects of single or repeated IV injection of 50 nm AuNRs and AuNSs on total WBCs and differential WBCs count

**Figure-2:**
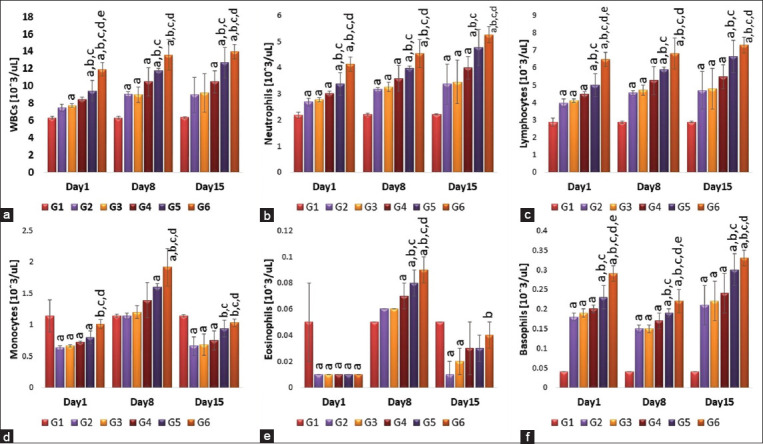
(a-f) Effect of single and repeated intravenous injection of 300 μg/kg body weight 50 nm either gold nanosphere or gold nanorods in male rabbits on total white blood cells (WBCs), differential WBCs on days 1, 8, and 15 post-treatment. Means carrying superscripts are significantly different at p<0.05 based on Tukey’s honestly significant difference. a=Significantly different versus G1, b=Significantly different versus G2, c=Significantly different versus G3, d=Significantly different versus G4, and e=Significantly different versus G5.

Results indicated that total WBCs, neutrophils lymphocyte, monocytes, and eosinophils counts were significantly (p<0.05) higher in the repeated injection of AuNRs group when compared to the single-dose AuNRs or the vaccinated group (Figure-2).

The basophils count showed no significant difference regarding the shape of AuNPs but repeated injection of AuNRs significantly (p<0.05) increased basophils count compared to the single dose, or the AuNSs, the control, and vaccinated group (Figure-2f).

### Effects of single or repeated IV injection of 50 nm AuNRs and AuNSs on lysozyme and nitric oxide

**Figure-3:**
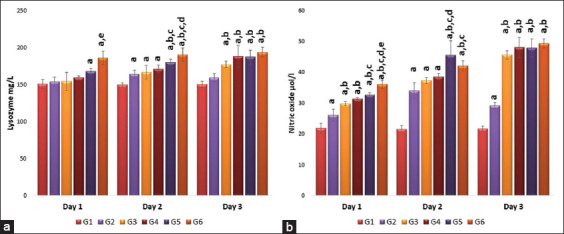
(a and b) Effect of single and repeated intravenous injection of 300 μg/kg body weight 50 nm either gold nanosphere or gold nanorods in male rabbits on lysozyme and nitric oxide on days 1, 2, and 3 post-treatment. Means carrying superscripts are significantly different at p<0.05 based on Tukey’s honestly significant difference. a=Significantly different versus G1, b=Significantly different versus G2, c=Significantly different versus G3, d=Significantly different versus G4, and e=Significantly different versus G5.

The effect of AuNP shape on lysozyme is demonstrated in Figure-3. Results indicated that on day 1 after repeated injection of AuNRs, the lysosome level was significantly (p<0.05) higher than G4, G5, and G2. Furthermore, repeated injection of AuNRs (G6) significantly increased NO level on day 1. While on day 2, a single injection of AuNRs significantly (p<0.05) increased NO levels compared to the other groups. On day 3, following single or repeated injection of AuNRs or AuNSs significantly (p<0.05) increased NO levels compared to control (G1) or vaccinated group (G2).

### Effects of single or repeated IV injection of 50 nm AuNRs and AuNSs on lymphocyte transformation on day 8

**Figure-4:**
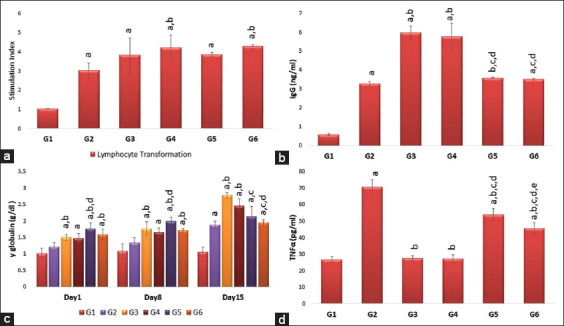
(a-d) Effects of single and repeated intravenous injection of 300 μg/kg body weight 50 nm either gold nanosphere or gold nanorods in male rabbits on γ-globulin, lymphocyte transformation on day 8 and immunoglobulin G and tumor necrosis factor-alpha on day 15. Means carrying superscripts are significantly different at p<0.05 based on Tukey’s honestly significant difference. a=Significantly different versus G1, b=Significantly different versus G2, c=Significantly different versus G3, d=Significantly different versus G4, and e=Significantly different versus G5.

Concerning the lymphocyte transformation on day 8, there was no significant difference among the different shapes or doses of AuNRs and AuNSs, but repeated injection of AuNRs significantly (p<0.05) increased lymphocytes transformation compared to the vaccinated group (Figure-4a).

### Effects of single or repeated IV injection of 50 nm AuNRs and AuNSs on IgG, γ-globulin, and TNF-α on day 15

Concerning the IgG, there was no significant difference regarding the different shapes; however, a significant (p<0.05) increase was recorded in G3 compared to G5 and G2 (Figure-4b). Furthermore, G4 showed a significant (p<0.05) increase compared to G6 and G2. The γ-globulin level also showed no significant difference regarding the shape or the dose of AuNPs on day 1 and day 8 of treatment; while on day 15 after treatment, repeated AuNRs injection significantly (1.95±0.09) decreased γ-globulin level when compared to the single dose (G4) and the vaccinated group (Figure-4). In terms of TNF-α on day 15, there was a significant difference regarding the shape and dose, where G2 showed a significant (p<0.05) higher value compared to G6, G3, and G5 (Figure-4).

### Effects of single or repeated IV injection of 50 nm AuNRs and AuNSs on comet assay parameters

**Figure-5:**
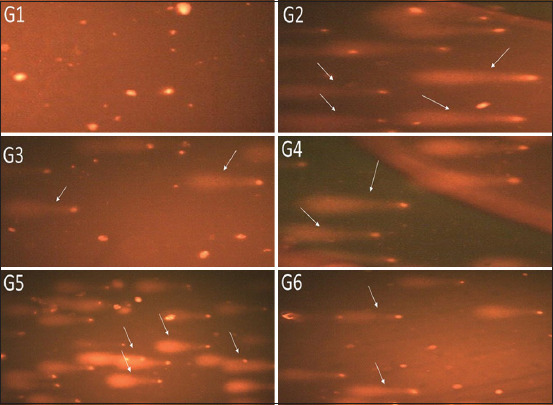
Effect of single and repeated intravenous injection of 300 μg/kg body weight 50 nm either gold nanospheres (AuNSs) or gold nanorods (AuNRs) in male rabbits on comet test parameters on day 8 post-treatment. It shows white blood cells with fluorescent DNA comets 8 days post-treatment. G1 represents the control negative (non-treated non-vaccinated) group with intact cells. G2 represents control positive (vaccinated only), which shows the highest degree of DNA damage with the longest tail. The same high degree DNA damage was in G5. On the contrary, G3 (AuNSs single IV dose) shows the lowest degree of DNA damage. On the other hand, in G4 and G6 (AuNRs single and repeated IV dose), little degree of DNA damage was seen. Some cells showing comets were marked with white arrows.

Table-1 represents the effects of nanoparticles on the comet assay. Blood sample analysis of DNA damage (after 8 days’ post-treatment) shows a statistically significant decrease in tail intensity (measurement of the extent of DNA damage) of samples of all treated groups in comparison to G2 (vaccinated group) samples (28.3±1.9). Moreover, regarding the shape, G6 shows a significant decrease in the degree of DNA damage (16.6±1.21) than G5 (20±1.17) and the G2. On the other hand, dose G3 (15±1.36) shows a significant decrease in the degree of DNA damage than G5 (Figure-5). Single dose injection of AuNRs significantly (p<0.05) increased in tail length compared to repeated AuNRs injection or the vaccinated group (Table-1). Regarding the % of DNA in the tail, there was a significant difference regarding the shape and dose, where G4 showed a significant (p<0.05) decrease compared to G3, G6, and G2 (Table-1). Concerning tail moment, there was a significant difference regarding the shape and dose, where G6 showed significantly (p<0.05) higher values of the tail moment compared to G4, G5, and G2 (Table-1). Furthermore, the olive tail moment was affected by shape and dose, where G6 showed a significantly higher (p<0.05) value compared to G4, G5, and G2 (Table-1).

**Table 1:** Effect of single and repeated intravenous injection of 300 µg/kg body weight of either 50nm gold nanosphere or gold nanorods in male rabbits on comet test parameters on day 8 post-treatment. Results are reported as Mean±SD (n=5).

Groups	Day 8				

	DNA damage (%)	Tail length pixels	Percent DNA in tail	Tail moment	Olive tail moment
G1	5.9±0.34	9.5±0.56	3.89±0.22	0.43±0.03	0.76±0.04
G2	28.3±1.90^a^	11.19±0.75^a^	3.30±0.22^a^	0.28±0.02^a^	0.44±0.03^a^
G3	15±1.36^a^, ^b^	8.13±0.74^b^	3.38±0.31^a^	0.25±0.02^a^	0.49±0.05^a^
G4	15.7±1.32^a^,^b^	14.71±1.23^a^, ^b^, ^c^	1.89±0.16^a^, ^b^, ^c^	0.27±0.02^a^	0.53±0.04^a^, ^b^
G5	20±1.17^a^, ^b^, ^c^, ^d^	8.5±0.50^b^, ^d^	3.49±0.20^d^	0.3±0.02^a^, ^c^	0.63±0.04^a^, ^b^, ^c^,^d^
G6	16.6±1.21^a^, ^b^, ^e^	13±0.95^a^, ^b^, ^c^,^d^, ^e^	3.48±0.25^d^	0.44±0.03 b, c, d, e	0.88±0.06^a^, ^b^, ^c^, ^d^, ^e^

Means carrying superscripts are significantly different at p*<*0.05 based on Tukey’s HSD=Honestly significant difference.

a Significantly different versus G1,

b Significantly different versus G2,

c Significantly different versus G3,

d Significantly different versus G4,

e Significantly different versus G5. SD=Standard deviation

**Figure-6:**
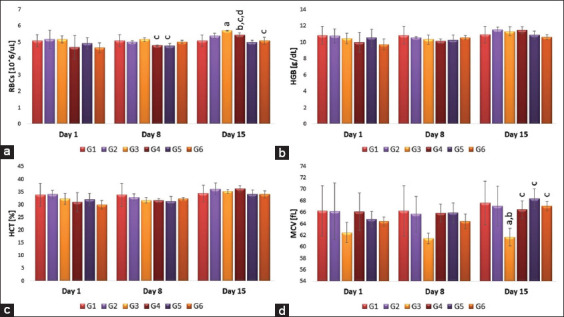
(a-d) Effects of single and repeated intravenous injection of 300 μg/kg body weight 50 nm either gold nanosphere or gold nanorods in male rabbits on red blood cells, hemoglobin, hematocrit, and mean corpuscular volume on days 1, 8, and 15 post-treatment. Means carrying superscripts are significantly different at p<0.05 based on Tukey’s honestly significant difference. a=Significantly different versus G1, b=Significantly different versus G2, c=Significantly different versus G3, d=Significantly different versus G4, and e=Significantly different versus G5.

**Figure-7:**
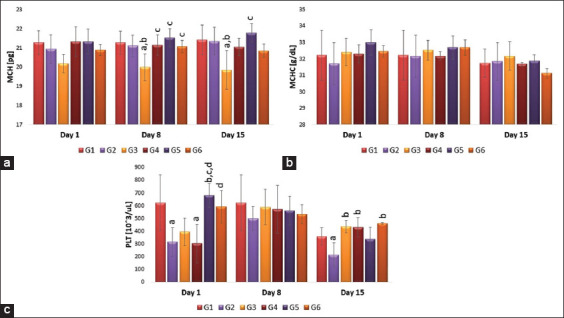
(a-c) Effects of single and repeated intravenous injection of 300 μg/kg body weight 50 nm either gold nanosphere or gold nanorods in male rabbits on mean corpuscular hemoglobin, mean corpuscular hemoglobin concentration, and PLT on days 1, 8, and 15 post-treatment. Means carrying superscripts are significantly different at p<0.05 based on Tukey’s honestly significant difference. a=Significantly different versus G1, b=Significantly different versus G2, c=Significantly different versus G3, d=Significantly different versus G4, and e=Significantly different versus G5.

The effect of the AuNPs on the hematological parameters is presented in Figures-6 and 7. The red blood cells (RBCs) count showed no significant difference in terms of shape and dose after 1 and 8 days of treatment compared to the vaccinated group, while on day 15 after treatment, G5 (repeated AuNSs) showed a significant (p<0.05) decrease in RBCs count compared to G3 (single- dose nanosphere) or vaccinated group (G2) (Figure-6a). Moreover, hemoglobin, hematocrit, and mean corpuscular hemoglobin concentration values did not significantly differ between groups regarding shape, single or repeated injection of AuNPs throughout the experiment (Figures-6b and c). In addition, mean corpuscular volume (MCV) values did not differ in shape and dose of AuNPs on days 1 and 8 post-treatment. While, on day 15 after treatment, there was a significant difference in terms of shape and dose where single-dose AuNSs (G3) showed a significantly (p<0.05) decrease in MCV compared to single-dose AuNRs (G4), repeated-dose nanosphere (G5), or vaccinated group (G2, Figure-7a). Furthermore, no significant difference was observed in terms of shape and dose of AuNPs in mean corpuscular hemoglobin (MCH) values on day 1 post-treatment; however, on days 8 and 15 post-treatment, there was a significant difference in terms of shape and dose, where single-dose AuNSs (G3) showed significantly (p<0.05) lower values compared to single-dose AuNRs (G4), repeated-dose AuNSs (G5), and vaccinated group (G2) (Figure-7b). Regarding the platelet count, on day 1 post-treatment, there was no significant difference in terms of the shape. However, a significant difference was observed regarding the dose where repeated-dose AuNSs (G5) showed a significant (p<0.05) increase in the PLT count compared to single-dose AuNSs (G3) and vaccinated group (G2). On day 8 post-treatment, no significant difference was observed regarding the shape or the dose of AuNPs. While, on day 15 post-treatment, repeated injection of AuNRs (G6) significantly (p<0.05) increased platelet count compared to the vaccinated group (G2), but there was no significant difference regarding the shape or dose between the other groups (Figure-7c).

**Figure-8:**
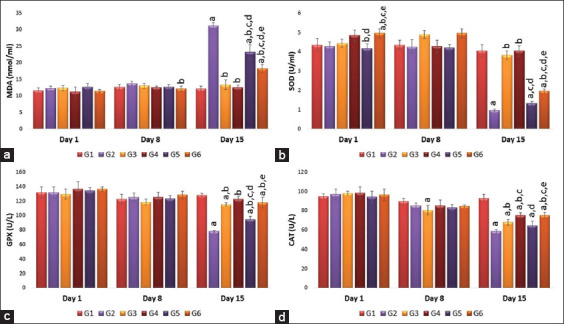
(a-d) Effects of single and repeated intravenous injection of 300 μg/kg body weight 50 nm either gold nanosphere or gold nanorods in male rabbits on malondialdehyde, superoxide dismutase, glutathione peroxidase, and catalase on days 1, 8, and 15 post-treatment. Means carrying superscripts are significantly different at p<0.05 based on Tukey’s honestly significant difference. a=Significantly different versus G1, b=Significantly different versus G2, c=Significantly different versus G3, d=Significantly different versus G4, and e=Significantly different versus G5.

The effects of AuNSs or AuNRs on MDA, SOD, GPx, and CAT are demonstrated in Figure-8. MDA levels showed no significant difference between treatment groups regarding the shape or the dose of gold particles compared to the vaccinated group (G2) on days 1 and 8 days of treatment. However, there was a significant difference regarding the shape and the dose on day 15 after treatment, where G6 showed a significant (p<0.05) decrease in MAD levels compared to G4, G5, and G2 (Figure-8a). Regarding the SOD levels, on days 1 and 15 post-treatment, there was a significant difference regarding the shape and the dose of AuNPs, where the repeated-dose AuNRs (G6) showed a significant (p<0.05) increase in SOD levels when compared to repeated AuNSs group (G5) or the vaccinated group (G2) (Figure-8b). Concerning GPx levels, on days 1 and 8 post-treatment, there was no significant difference between the treatment groups and the vaccinated group in shape and dose. However, on day 15 post-treatment, there was a significant difference regarding the shape and the dose where repeated-dose AuNSs (G5) showed a significant (p<0.05) decrease in GPx concentration compared to single AuNSs (G3), repeated AuNRs (G6), and the vaccinated group (G2) (Figure-8c). Furthermore, on days 1 and 8 in CAT levels, there was no significant difference among the treatment groups in terms of shape or dose compared to the vaccinated group, while on day 15 post-treatment, there was a significant difference regarding the shape where single and repeated doses of AuNRs (G4 and G6) showed significantly (p<0.05) higher CAT levels than single or repeated AuNSs (G3 and G5) or the vaccinated group (G2). However, there was no difference regarding the dose (Figure-8d).

## Discussion

AuNPs have been linked to many biomedical applications, including genomics, immunological analysis, clinical chemistry, cancer imaging and therapy, and other fields. The ability of researchers to validate the safety of AuNPs for therapeutic application is critical to their widespread adoption [12]. However, it has been shown that the side effects and possible toxicity of AuNPs are highly related to their features, such as size, shape, and surface chemistry [30]. Hence, determining the most appropriate shape of AuNPs is necessary before their applications in clinical trials.

The blood and the AuNP have an immediate and crucial biological interaction because most AuNPs are intended to use through systemic IV usage. Plasma proteins and cells make up blood; when these components interact with AuNPs, they can have downstream effects that modify their medicinal impact [31].

This study estimated the effect of 50 nm AuNRs and AuNSs on the WBCs and the other hematological parameters. The results showed no significant difference in the WBCs count, lymphocytes, monocytes, eosinophils, and basophils in the treatment groups compared to the control group, except the repeated-dose AuNRs compared to AuNSs. The neutrophilic count was significantly increased by the repeated-dose AuNRs on day 1 post-treatment. These results were in line with Bashandy *et al*. [32] in terms of the effect of AuNPs where it was found that, in comparison to the control group, the AuNPs administered group demonstrated a slight increase in total leukocytic count along with substantial mild absolute neutrophils on the 3^rd^ day and considerable monocytosis on the 3^rd^, 5^th^, and 7^th^ days. However, there were differences in terms of the shape where the AuNPs were spherical. Zhang *et al*. [13] and Das *et al*. [33] also reported that the AuNPs showed a non-significant difference in the leukocytic count compared to the control group. The increased WBCs count could be attributed to that AuNRs in the blood were often recognized as foreign bodies, as WBCs defend invaders by phagocytosis germs or foreign bodies and producing antibodies to prevent infection. This ensures that AuNPs come into direct contact with leukocytes and increases the likelihood of AuNPs accumulation inside the WBCs [34].

Furthermore, the increased release of various cytokines, including pro-inflammatory and inflammatory cytokines, generated by the nanoparticles could explain the increase in neutrophil count Bashandy *et al*. [32]. That higher neutrophil count contrasts with the results by Sung *et al*. [35] which could be attributed to the size and/or the dose of the AuNPs. In addition, the significant monocytosis observed in the AuNPs administered group is mostly due to the involvement of monocytes in clearing the circulation of foreign materials [36]. Regarding the eosinophilic and the basophilic count, there was a significant increase by the repeated-dose AuNRs, and this disagreed with Bashandy *et al*. [32]. In contrast, i.p. injection of 50 nm AuNRs was associated with marked changes in blood profile in male rats [37]. This difference could be attributed to the route of AuNRs administration or the protocol used for treatment.

The treatment with the repeated dose of AuNRs also revealed a significant increase in the lysozymes and the nitric oxide activity after 3 days of treatment. Hence, AuNRs might enhance the nitric oxide release which is crucial in several biological functions [38]. Smaller AuNPs were trapped in the cytoplasmic vesicles and lysosomes of Kupffer cells, macrophages, the spleen, and the mesenteric lymph node in mice [39]. The γ-globulin levels showed a non-significant difference among groups except after 15 days where the single-dose AuNSs was significantly increased compared with the other groups. This contrasts with the findings of Bashandy *et al*. [32].

Inflammation is one probable cell response to NP-induced oxidative stress, which is why TNF-α was investigated. Regarding the inflammatory and immunological effect, single- and repeated-dose AuNPs (especially AuNRs) non-significantly affected the IgG levels but a single dose of AuNSs significantly increased IgG levels. On the other hand, TNF-α was significantly decreased. In concomitant, AuNPs are biocompatible, non-cytotoxic, and non-immunogenic. They decrease the generation of reactive oxygen species (ROS) and do not cause the pro-inflammatory cytokines, including TNF-α, to be elaborated [40]. On the contrary, Yen *et al*. [41] reported that with AuNPs treatment, the number of macrophages was reduced, and their size increased and followed by increased production of interleukin (IL)-1, IL-6, and TNF-α. Furthermore, AuNPs appear to enhance downstream IgG secretion [12,42]. This discrepancy might be due to the shape, dose, or type of injection (acute or chronic) of AuNPs.

The study of AuNPs’ genotoxicity is important because DNA damage can result in persistent mutations and/or genomic instability, leading to uncontrolled cell proliferation (which can lead to cancer) or cell death [43]. The genotoxicity of AuNPs was assessed in the present study, and the findings revealed that all types of AuNPs induced DNA damage as determined by the catechol-O-methyltransferase assay. A dose-dependent rise in DNA damage induction was found over time (repeated dose of treatment) following AuNPs exposure, according to a time series of DNA damage induction (on day 8 post-treatment). These results were in agreement with May *et al*. [42]. The weakest DNA damaging effect was observed for single-dose AuNSs and AuNRs and repeated-dose AuNRs. The genotoxic potential of AuNPs is ranked, with repeated dosage AuNSs being the most genotoxic, followed by repeated-dose AuNR, single-dose AuNR, and single-dose AuNSs.

Furthermore, in this study, the highest increase in the olive moment was recorded in repeated-dose of AuNRs. These results are concomitant with Hassan *et al*. [44]. This effect could be attributed to the size of the AuNPs used in the present study (50 nm) that had a significant role in the toxicity. Because of their strong capability to penetrate cell membranes, diameters of 30-50 nm are considered the most hazardous compared to other sizes [45]. In addition, the accumulation of AuNPs within the lysosome activates the autophagosome mechanism, resulting in cell death [46]. Furthermore, the AuNPs spherical shape makes them 5 times more toxic than other shapes [47].

The hematological examination of AuNR and nanosphere treatment in the current study revealed a significant decrease in the RBCs count, MCV, and MCH after 15 days of treatment with single-dose AuNSs, indicating small-sized RBCs, while AuNR showed no significant change. These results completely agreed with Zhang *et al*. [13]. On the contrary to Bashandy *et al*. [32] found that at different time intervals, AuNPs administration to rabbits did not exhibit any significant change in all of the analyzed blood parameters compared to those of the control group. It was found that because of the interaction between AuNPs and the RBCs’ membrane, AuNPs influenced RBCs’ deformability and oxygen delivery ability [48].

The platelet count was significantly increased with repeated-dose AUnR after 15 days of treatment. This might indicate that repeated injection of AuNR could stimulate a coagulative effect [49]. This was in harmony with the findings of the study by Barathmanikanth *et al*. [50]. It was suggested that the injected AuNPs circulate in the blood mostly in particulate appearances; so, there is a potential existence of interaction of AuNPs with blood constituents and cells to stimulate a coagulative effect. In addition, injection of AuNR over 15 days showed that AuNPs at smaller concentrations did not result in any considerable toxicity or vascular reactions [49].

Multiple studies have shown that AuNPs have an antioxidant effect that significantly reduces the oxidative stresses that make ROS [51]. Lipid peroxidation is a process under which oxidants such as free radicals attack the lipids which contain carbon-carbon double bond(s), particularly polyunsaturated fatty acids. MDA is one of the end products of the peroxidation of polyunsaturated fatty acids within the cells. The high free radicals’ levels result in the overproduction of MDA, and its level is a standard marker of oxidative stress and the antioxidant state among patients with cancers [52]. In addition, SOD, GPx, and CAT are antioxidant enzymes that protect the body from the intracellular damage that can be caused by the oxidative free radical [53].

In this study, no significant change in oxidative stress parameters was induced after single-dose AuNRs injection, evidenced by the significant decrease in MDA levels and the significant increase of the SOD, GPx, and CAT levels after 15 days of treatment, compared to the treatment by AuNSs. Hence, the AuNRs inhibited the elevated ROS generation and lipid peroxidation, thus restoring the antioxidant system to normal. Similar results were previously reported in rats [54,55], where AuNPs of 10-50 nm diameters did not show any oxidative stress. Furthermore, Leonavičienė *et al*. [56] reported that injection of 50 nm of AuNPs reduced the MDA production and significantly enhanced the activity of the CAT enzyme, which was considered a principal antioxidant responsible for the direct eradication of ROS produced [56,57]. In contrast, direct exposure of hepatocytes to AuNRs depleted the GSH levels, increased lipid peroxidation, caspase-3 expression, and inducing oxidative stress [58]. This difference could be due to the experimental design, route, shape, or dose of AuNPs injected.

## Conclusion

The present study demonstrates for the 1^st^ time the effect of the shape of AuNPs on immune response in rabbits. From the findings of this study, it is concluded that the effect of AuNPs is shape and dose-dependent. The IV repeated-dose treatment with 50 nm AuNRs over 15 days induced a significant antioxidant effect, with no considerable toxicity or vascular reactions. AuNPs in the form of 50 nm or AuNRs at 300 μg/kg BWT concentrations are safe for therapeutic use.

## Authors’ Contributions

ETM, BSAK, DMA, ASSA, SME, MBM, and MMA: Designed and performed the work, did the statistical analysis, and drafted the manuscript. ASSA: Prepared the AuNPs. ASSA: Wrote the manuscript. All authors read and approved the final manuscript.

## References

[1] Jassim A.M.N, Al-Kazaz F.F.M, Kamel L.A, Farhan S.A, Noori hO.M (2015). Biochemical study for gold and silver nanoparticles on thyroid hormone levels in sera of patients with chronic renal failure. J. Pharm. Chem. Biol. Sci.

[2] Kumar A, Mazinder Boruah B, Liang X.J (2011). Gold nanoparticles:Promising nanomaterials for the diagnosis of cancer and HIV/AIDS. J. Nanomater.

[3] Glazer E.S, Zhu C, Hamir A.N, Borne A, Thompson C.S, Curley S.A (2011). Biodistribution and acute toxicity of naked gold nanoparticles in a rabbit hepatic tumor model. Nanotoxicology.

[4] Ho J.A.A, Chang H.C, Shih N.Y, Wu L.C, Chang Y.F, Chen C.C, Chou C (2010). Diagnostic detection of human lung cancer-associated antigen using a gold nanoparticle-based electrochemical immunosensor. Anal. Chem.

[5] Shankar S.S, Bhargava S, Sastry M (2005). Synthesis of gold nanospheres and nanotriangles by the Turkevich approach. J. Nanosci. Nanotechnol.

[6] Bloise N, Massironi A, Della Pina C, Alongi J, Siciliani S, Manfredi A, Visai L (2020). Extra-small gold nanospheres decorated with a thiol functionalized biodegradable and biocompatible linear polyamidoamine as nanovectors of anticancer molecules. Front. Bioeng. Biotechnol.

[7] Khan H.A, Abdelhalim M.A.K, Al-Ayed M.S, Alhomida A.S (2012). Effect of gold nanoparticles on glutathione and malondialdehyde levels in liver, lung, and heart of rats. Saudi J. Biol. Sci.

[8] Pal R, Panigrahi S, Bhattacharyya D, Chakraborti A.S (2013). Characterization of citrate capped gold nanoparticle-quercetin complex:Experimental and quantum chemical approach. J. Mol. Struct.

[9] Giljohann D.A, Seferos D.S, Daniel W.L, Massich M.D, Patel P.C, Mirkin C.A (2010). Gold nanoparticles for biology and medicine. Angew. Chem. Int. Ed. Engl.

[10] Roy K, Kanwar R, Kanwar J (2015). Molecular targets in arthritis and recent trends in nanotherapy. Int. J. Nanomed.

[11] Abdoon A.S.S, Al-Ashkar E.A, Kandil O.M, Shaban A.M, Khaled H.M, El Sayed M.A, El Shaer M.M, Shaalan A.H, Eisa W.H, Eldin A.A.G, Hussein H.A, El Ashkar M.R, Ali M.R, Shabaka A.A (2016). Efficacy and toxicity of plasmonic photothermal therapy (PPTT) using gold nanorods (AuNRs) against mammary tumors in dogs and cats. Nanomed. Nanotechnol. Biol. Med.

[12] Dykman L.A, Khlebtsov N.G (2017). Immunological properties of gold nanoparticles. Chem. Sci.

[13] Zhang X.D, Wu H.Y, Di Wu Y.W, Chang J.H, Zhai Z.B, Meng A.M, Liu P.X, Zhang L.A, Fan F.Y (2010). Toxicologic effects of gold nanoparticles *in vivo* by different administration routes. Int. J. Nanomed.

[14] Murphy C.J, Thompson L.B, Chernak D.J, Yang J.A, Sivapalan S.T, Boulos S.P, Huang J, Alkilany A.M, Sisco P.N (2011). Gold nanorod crystal growth:From seed-mediated synthesis to nanoscale sculpting. Curr. Opin. Coll. Interface Sci.

[15] Frens G (1973). Controlled nucleation for the regulation of the particle size in monodisperses gold suspensions. Nat. Phys. Sci.

[16] Schultz L.A (1987). Method in Clinical Chemistry. The C. V. Mosby Cost Louis, United States.

[17] Boyum A (1968). Isolation of mononuclear cell and granulocytes from human blood. Scand. J. Clin. Lab. Invest.

[18] Hudson L, Hay F.C (1980). Immunology.

[19] Rai-El-Balahaa G, Pellerin J.L, Bodin G, Abdullah H.A, Hiron H (1985). Lymphoblastic transformation assay of sheep peripheral blood lymphocytes:A new rapid and easy to read techniques. Comp. Immun. Microbiol. Infec. Dis.

[20] Denise I, Bounous A, Raymond Q, Campagnoli A, John Brown B (1992). Comparison of MTT colorimetric assay and tritiated thymidine uptake for lymphocyte proliferation assay using chicken splenocytes. Avian Dis.

[21] Erhard M.H, Quistrop I, Von Schrmner I, Jungling A, Kaspers B, Schmidt P, Kuhmann R (1992). Development of a specific enzyme-linked immunosorbent antibody assay for detection of immunoglobulins G, M, and A. Poultry Sci.

[22] Daves C (1971). Diagnostic value of muramidase. Postgrad. Med.

[23] Olaniyan M.F, Atibor R.A, Afolabi T (2018). Evaluation of tumor necrosis factor-alpha (TNFa), interleukin 4, interleukin 6, aspartate aminotransferase, and alanine aminotransferase in rabbits overdosed with ibuprofen and supplemented with guava leaf (*Psidium guajava*) extract. Biomed. Biotechnol. Res. J.

[24] Singh N.P, McCoy M.T, Tice R.R, Schneider E.L (1988). A simple technique for quantitation of low levels of DNA damage in individual cells. Exp. Cell Res.

[25] Ramadan A.A, Attia E.R.M (2003). Natural Killing Molecules in Cervical Mucus of Buffaloes During Estrous Cycle.

[26] Ohkawa H, Ohishi N, Yagi K (1979). Assay for lipid peroxides in animal tissues by thiobarbituric acid reaction. Anal. Biochem.

[27] Aebi H (1984). Catalase *in vitro*. Methods Enzymol.

[28] Pascual P, Martinez-Lara E, Barcena J.A, López-Barea J, Toribio F (1992). Direct assay of glutathione peroxidase activity using high-performance capillary electrophoresis. J. Chromatogr. B Biomed. Sci. Appl.

[29] Nishikimi M, Rao N.A, Yagi K (1972). The occurrence of superoxide anion in the reaction of reduced phenazine methosulfate and molecular oxygen. Biochem. Biophys. Res. Commun.

[30] Daraee H, Eatemadi A, Abbasi E, Fekri Aval S, Kouhi M, Akbarzadeh A (2016). Application of gold nanoparticles in biomedical and drug delivery. Artif. Cells Nanomed. Biotechnol.

[31] Shah N.B, Bischof J.C (2013). Blood protein and blood cell interactions with gold nanoparticles:The need for *in vivo* studies. Bionanomaterials.

[32] Bashandy M.M, Ahmed A.R, El-Gaffary M, Abd El-Rahman S.S (2015). Gold nanoparticle:Synthesis, characterization, clinicopathological, pathological, and bio-distribution studies in rabbits. Int. J. Biol. Biomol. Agric. Food Biotechnol. Eng.

[33] Das S, Debnath N, Mitra S, Datta A, Goswami A (2012). Comparative analysis of stability and toxicity profile of three differently capped gold nanoparticles for biomedical usage. Biometals.

[34] He Z, Li C, Zhang X, Zhong R, Wang H, Liu J, Du L (2018). The effects of gold nanoparticles on the human blood functions. Artif. Cells Nanomed. Biotechnol.

[35] Sung J.H, Ji J.H, Park J.D, Song M.Y, Song K.S, Ryu H.R, Yoon J.U, Jeon K.S, Jeong J, Han B.S, Chung Y.H, Chang H.K, Lee J.H, Kim D.W, Kelman B.J, Yu I.J (2011). Subchronic inhalation toxicity of gold nanoparticles. Particle Fibre Toxicol.

[36] Weiss D.J, Wardrop K.J (2011). Schalm's Veterinary Hematology.

[37] Said M.A, Abdoon A.S.S, Said A.A, Shams G, Elnabtity S.M (2019). Acute and chronic hematological study of gold nanorods in rats. Res. J. Pharm. Biol. Chem. Sci.

[38] Silva B.R, Lunardi C.N, Araki K, Biazzotto J.C, Da Silva R.S, Bendhack L.M (2014). Gold nanoparticle modifies nitric oxide release and vasodilation in rat aorta. J. Chem. Biol.

[39] Cho W.S, Cho M, Jeong J, Choi M, Han B.S, Shin H.S, Cho M.H (2010). Size-dependent tissue kinetics of PEG-coated gold nanoparticles. Toxicol. Appl. Pharmacol.

[40] Shukla R, Bansal V, Chaudhary M, Basu A, Bhonde R.R, Sastry M (2005). Biocompatibility of gold nanoparticles and their endocytotic fate inside the cellular compartment:A microscopic overview. Langmuir.

[41] Yen H, Hsu S.H, Tsai C.L (2009). Cytotoxicity and immunological response of gold and silver nanoparticles of different sizes. Small.

[42] May S, Hirsch C, Rippl A, Bohmer N, Kaiser J.P, Diener L, Wick P (2018). Transient DNA damage following exposure to gold nanoparticles. Nanoscale.

[43] Hoeijmakers J.H (2009). DNA damage, aging, and cancer. N. Engl. J. Med.

[44] Hassan Z.A, Obaid H.H, Al-Darraji M.N (2020). *In vivo* genotoxicity assessment of gold nanoparticles of different doses by comet assays. Indian J. Forensic Med. Toxicol.

[45] Wu M, Guo H, Liu L, Liu Y, Xie L (2019). Size-dependent cellular uptake and localization profiles of silver nanoparticles. Int. J. Nanomed.

[46] Su S.S, Chang I (2018). Review of production routes of nanomaterials. In:Commercialization of Nanotechnologies-a Case Study Approach. Springer, Berlin, Germany.

[47] Cordani M, Somoza Á (2019). Targeting autophagy using metallic nanoparticles:A promising strategy for cancer treatment. Cell. Mol. Life Sci.

[48] He Z, Liu J, Du L (2014). The unexpected effect of PEGylated gold nanoparticles on the primary function of erythrocytes. Nanoscale.

[49] Lee J.H, Gulumian M, Faustman E.M, Workman T, Jeon K, Yu I.J (2018). Blood biochemical and hematological study after subacute intravenous injection of gold and silver nanoparticles and coadministered gold and silver nanoparticles of similar sizes. BioMed Res. Int.

[50] Barathmanikanth S, Kalishwaralal K, Sriram M, Pandian S.R.K, Youn H.S, Eom S, Gurunathan S (2010). Antioxidant effect of gold nanoparticles restrains hyperglycemic conditions in diabetic mice. J. Nanobiotechnol.

[51] Oueslati M.H, Tahar L.B, Harrath A.H (2020). Catalytic, antioxidant and anticancer activities of gold nanoparticles synthesized by kaempferol glucoside from Lotus leguminosae. Arabian J. Chem.

[52] Ayala A, Muñoz M.F, Argüelles S (2014). Lipid peroxidation:Production, metabolism, and signaling mechanisms of malondialdehyde and 4-hydroxy-2-nonenal. Oxid. Med. Cell. Longev.

[53] Ighodaro O.M, Akinloye O.A (2018). First line defence antioxidants-superoxide dismutase (SOD), catalase (CAT) and glutathione peroxidase (GPX):Their fundamental role in the entire antioxidant defence grid. Alex. J. Med.

[54] Hassan A.A, Abdoon A.S.S, Elsheikh S.M, Khairy M.H, Eldin A. A. G, Elnabtity S.M (2019). Effect of acute gold nanorods on reproductive function in male albino rats:Histological, morphometric, hormonal, and redox balance parameters. Environ. Sci. Pollut. Res.

[55] Orabi S.H, Mansour D.A, Fathalla S. I, Gadallah S.M, Eldin A.G, Abdoon A.S (2019). Effects of administration of 10 nm or 50 nm gold nanoparticles (AuNPs) on blood profile, liver and kidney functions in male albino rats. Indian J. Biochem. Biophys.

[56] Leonavičienė L, Kirdaitė G, Bradūnaitė R, Vaitkienė D, Vasiliauskas A, Zabulytė D, Ramanavičienė A, Ramanavičius A, Ašmenavičius T, Mackiewicz Z (2012). Effect of gold nanoparticles in the treatment of established collagen arthritis in rats. Medicina.

[57] Sul O.J, Kim J.C, Kyung T.W, Kim H.J, Kim Y.Y, Kim S.H, Kim J.S, Choi H.S (2010). Gold nanoparticles inhibited the receptor activator of nuclear factor-kb ligand (RANKL)-induced osteoclast formation by acting as an antioxidant. Biosci. Biotechnol. Biochem.

[58] Lingabathula H, Yellu N (2016). Cytotoxicity, oxidative stress, and inflammation in human Hep G2 liver epithelial cells following exposure to gold nanorods. Toxicol. Mech. Methods.

